# Electromagnetic Performances Analysis of an Ultra-wideband and Flexible Material Antenna in Microwave Breast Imaging: To Implement A Wearable Medical Bra

**DOI:** 10.1038/srep38906

**Published:** 2016-12-23

**Authors:** Ashiqur Rahman, Mohammad Tariqul Islam, Mandeep Jit Singh, Salehin Kibria, Md. Akhtaruzzaman

**Affiliations:** 1 Department of Electrical, Electronics and Systems Engineering, Faculty of Engineering and Built Environment, Universiti Kebangsaan Malaysia, Bangi, 43600, Selangor, Malaysia; 2 Laboratory of Spacecraft Environment Interaction Engineering, Kyushu Institute of Technology, 1-1 Sensui Tobata-ku, Kitakyushu, 804-8550, Japan; 3 Solar Energy Research Institute, Universiti Kebangsaan Malaysia, Bangi, 43600, Selangor, Malaysia

## Abstract

In this paper, we report a compact and ultra-wide band antenna on a flexible substrate using the 5-(4-(perfluorohexyl)phenyl)thiophene-2-carbaldehyde compound for microwave imaging. In contrast to other microwave based imaging systems, such as an array of 16 antennas, we proposed a bi-static radar based imaging system consisting of two omnidirectional antennas, which reduces complexity and the overall dimension. The proposed compact antennas are 20 × 14 mm^2^ and designed for operating at frequencies from 4 to 6 GHz. To allow for implantation into a bra, the electromagnetic performances of the antennas must be considered in bending conditions. In comparison with the recently reported flexible antennas, we demonstrated both electromagnetic performance and imaging reconstruction for bending conditions. For the proof of concept, the electromagnetic performances both at flat and bending conditions have been verified using a homogeneous multilayer model of the human breast phantom. Our results demonstrate that the antenna, even at bending conditions, exhibits an excellent omni-directional radiation pattern with an average efficiency above 70% and average gain above 1 dBi, within the operational frequency band. The comprehensive aim of the realized antenna is to design a biodegradable and wearable antenna-based bra for early breast cancer detection in the future.

Breast cancer, which develops from breast tissue, is one of the crucial health issues in the world today. Breast cancer is one of the most widespread cancers in women globally, accounting for 25% of all cases^1^. The most common symptoms of this cancer is noticing a lump that is different from typical breast tissue, and in more than 80% cases, women can feel such lumps by themselves^2^. The world health organization reported 1.68 million cases and 522,000 deaths up to 2012 from breast cancer^1^. Early detection can be the key factor to increasing the survival rate, which accelerates the urgent requirement for a trustworthy, comfortable and highly efficient technique for early breast cancer detection.

Presently, X-ray mammography is the conventional technique for screening and detecting breast cancer. Unfortunately, the rate of failure in detecting the tumour is still quite high and varies from 4% to 34%^3^. Moreover, it is evident that ionization caused by X-ray mammography represents a severe health threat, and there is even a chance of women developing cancer from such an examination method^4^,^5^. Patients are also unwilling to endure uncomfortable breast compression during this diagnosis technique. As an alternative, magnetic resonance imaging (MRI) draws attention to detect breast cancer because of its unique feature of high sensitivity; however, the examination is very expensive and has little specificity, which can lead to an incorrect diagnosis^6^,^7^. Ultrasound (US) is an alternative detection method, with a 17% false-negative rate^8^. To improve the detection system, combinations of different models have also been investigated^8^. The statistics presented in Table 1 confirmed that even a combination of different models are still not adequate for cost reduction, safety improvement, prevention of discomfort and more importantly, an increase in accuracy. Such limitations motivate researchers around the globe to explore new alternative diagnosis methods.

Breast cancer detection through electromagnetic (EM) radiation earned immense attention in recent years. The detection of a breast cancer tumour is a biomedical effort, and this application corresponds to microwave tomography. The technique of microwave imaging (MWI) is a nonionizing and cost effective approach with sufficient penetration into breast tissue for breast cancer tumour detection. The EM detection technique depends on the dielectric and electromagnetic properties, where the permittivity, conductivity and electrical parameters of cancerous tissue is 5 to 10 times larger than those of normal breast tissue^9^.

In MWI, the antenna acts as transmitting and receiving sensors, where the transmitting antenna illustrates the breast through microwave signals, whereas scattered signals from the breast tissue are collected by the receiving antenna. Because of the different dielectric permittivity and conductivity between cancerous tissue and normal tissue, the incident wave scatters differently from cancerous tissue, thereby indicating its presence. In recent years, microwave imaging has earned immense interest in several clinical investigations; microwave imaging can detect tumours with minimum size of 5–10 mm at 80–90% accuracy^10^.

The first reported near-field microwave imaging method by Meaney *et al*.^11^ achieved at Dartmouth College. The system was comprised of 32 monopole antennas in a circular array arrangement with a frequency range from 300–1000 MHz. Later, researchers from the University of Bristol developed an ultra-wideband (UWB) microwave imaging system operated at frequencies from 4.5–10 GHz utilizing the cavity-backed patch antennas^12^. Extensive studies have been conducted to develop a microwave imaging system that utilizes dielectric resonator antennas, dipole antennas slot antennas, patch antennas, MEMS-steerable antennas, Horn antennas and Vivaldi antennas^13^. The aforementioned antennas have some limitations, such as the requirement of matching liquid, thereby increasing the complexity and the antenna volume as well as creating discomfort for patients and incompatibility in the array.

There are mainly two techniques in MWI; microwave tomography and a radar based technique. In microwave tomography, the electrical properties of the breast were reconstructed by calculating a nonlinear and ill-posed inverse scattering signal. In contrast, in the radar-based technique, different back scattered signals caused by the differences in dielectric properties were used to map the breast tissue. In both cases, physical measurements can be examined in either the time domain or the frequency domain, but the frequency domain method is superior to time domain because of the poor signal-to-noise ratio (SNR) exhibited in the time domain.

Currently, multi-static and mono-static radar based systems are used for breast cancer detection. The multi-static radar system uses an antenna array and a complex switch network controller^14^,^15^. The antenna array normally consists of more than four antennas to collect a large number of transmission coefficients and thus obtain images of high resolution. The problem is that the geometrical dimensions of the utilized antennas must be as small as possible to maximize the number of antennas employed in the array. This, in turn, will result in a higher working frequency band, which lacks the required penetration of electromagnetic energy into the breast. Moreover, a lower frequency signal can penetrate deeper into the tissue to enhance the accuracy, but it provides low resolution images. A multi-static radar method can improve this resolution with the cost of complex circuitry and switch matrix. In contrast, the mono-static or bi-static radar system^16^ using a single antenna or an antenna pair and higher frequency above 4 GHz can provide high resolution and accurate data with mechanical movement. Hence, the design of the antenna is only concerned with the gain, bandwidth, efficiency and radiation pattern.

In the last decade body area network^17^, health monitoring^18^ and some small broadband antennas for breast cancer detection have been presented. In recent years, planar monopole antennas^19^,^20^ have been drawing huge interest because of their unique features with small dimension, ease of fabrication, plain structures and wideband behaviour; however, these antennas are rigid rather flexible, which is the prerequisite for wearable device. To the best of our knowledge, Hadi *et al*.^15^ recently reported the first flexible antenna array for breast cancer detection, although microwave imaging and electromagnetic performances were not reported. This year, another flexible antenna for microwave imaging was reported by Porter *et al*.^13^, although the electromagnetic performance (efficiency, gain and radiation pattern) of such antennas were not reported.

In this report, we deployed a bi-static radar system for breast cancer detection. We designed a compact, highly efficient and omnidirectional antenna fabricated on a flexible and biodegradable organic material, as shown in Fig. 1(a). Our research focuses on the following:

- Experimental characterization of microwave dielectric properties of the organic material.
- Comprehensive numerical analysis of the electromagnetic performance, including the reflection coefficient, efficiency, gain and radiation pattern for both flat and bending conditions.
- Image processing and comparison between flat and bending conditions.

The long-term plan of this research is to design a wearable and biodegradable antenna-based bra for breast scanning to detect cancerous tissue at the early stage. The mechanical flexibility and adequate electromagnetic performances at bending conditions signify this antenna as a promising candidate for use as a future wearable and biodegradable antenna for breast scanning.

## Microwave Dielectric Characterization

**Figure 1:**
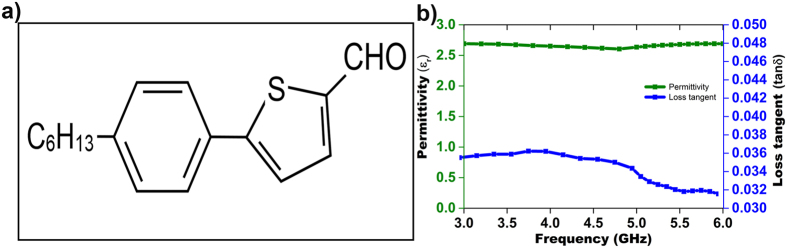
(**a**) Chemical structure of organic material and (**b**) microwave dielectric properties of organic material.

In this study, to validate the flexible organic material as a substrate, we utilize a dielectric measurement kit (DAK 3.5) coupled with a network analyser (PNA 85070E) to measure the dielectric properties of the substrate material from 3 to 6 GHz. The complete measurement technique, controlled by a network analyser, was used to measure the response of the material to RF or microwave signals by measuring the transmitted wave through the material under test (MUT). Figure 1(b) reveals that the permittivity and loss tangent of the material slightly fluctuates with the applied frequency. Such fluctuation could be explained according to the Maxwell–Wagner model of interfacial polarization and Koop’s phenomenological theory^21^,^22^. In addition, Debye clarified that because of the persistence of interfacial polarization, the relaxation process extends, thereby increasing the relaxation time compared to the dipole polarization^23^. The measured dielectric permittivity is 2.64 throughout the frequency band.

The characteristics, such as loss tangent (tan *δ*), impedance bandwidth and efficiency of an antenna, are correlated and must be optimized collectively. The quality factor of the antenna is related to the surface wave loss (*Qsw*), dielectric loss (*Qd*) and radiation loss (*Qrad*). The loss caused by the surface waves are negligible when the substrate is very thin (*h* ≪ *λ*_*o*_). From the material perspective, the dielectric loss is the key parameter and can be expressed by ref. 24:


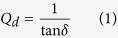


where tan *δ* denotes loss tangent of the substrate material. There are two types of losses in dielectric materials, such as conduction loss caused by the flow of electron charges and a dielectric loss caused by the atom movement in an alternating field. Among the total core loss, dielectric loss plays a significant function in microwave dielectric materials. The ratio of the lossy reaction of the electric field *E* to the lossless reaction in the curl equation is defined as loss tangent and is less than 90°. The measured loss tangent of the utilized organic substrate is 0.03, as shown in Fig. 1(b). A low loss tangent helps to reduce the dielectric loss, which improves the efficiency of the electromagnetic radiator.

## Antenna Design

In this paper, an ultra-wideband monopole antenna with omnidirectional radiation pattern is designed for microwave imaging. The UWB antenna can be defined as an antenna transmission for which emitted signal bandwidth exceeds the lesser of 500 MHz or 20% of fractional bandwidth as follows^24^:


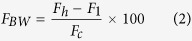


**Figure 2:**
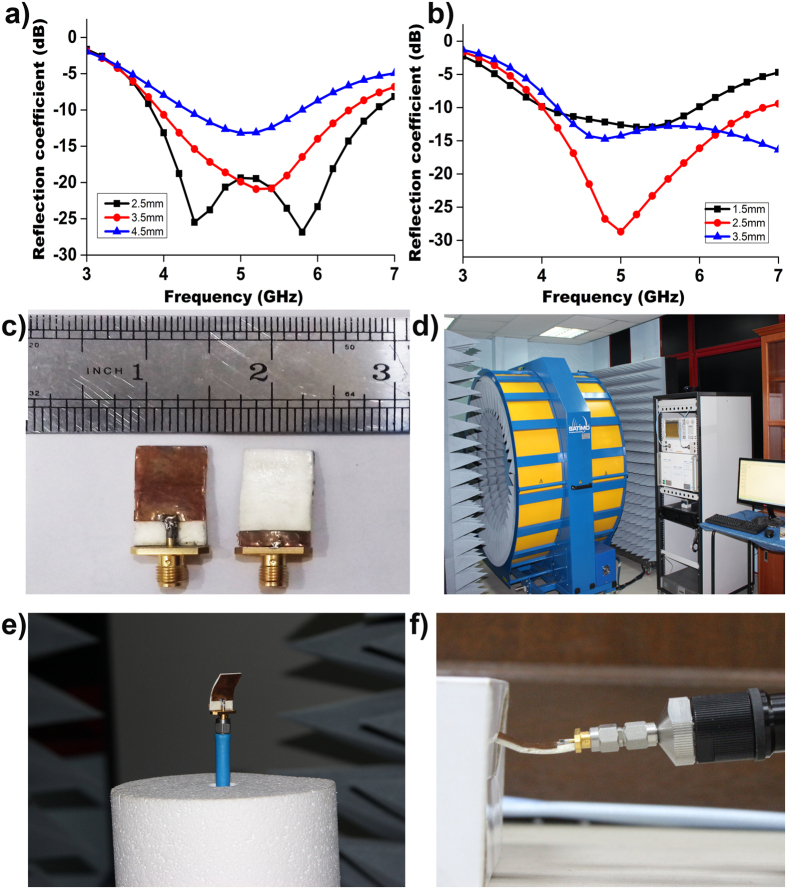
(**a**) Reflection coefficient response to the parametric change in the ground plane width; (**b**) reflection coefficient response to the parametric change in the transmission line width; (**c**) prototype of the proposed flexible antenna; and (**d**) SATIMO Star-lab setup (**e**) electromagnetic performances at bending conditions at the SATIMO Star-lab; (**f**) reflection coefficient measurement at bending conditions.

The width of the transmission line (*wf*) and the partial ground plane (*wg*) have a significant effect on increasing the impedance bandwidth. The ground plane and the transmission line that maintain a gap (*h*) obtain a suitable impedance matching^25^. Figure 2(a) demonstrates that the ground plane of an adjusted value of 14 × 3.5 mm^2^ results in a widest impedance bandwidth with better reflection coefficient when maintaining a gap of 2.5 mm between the transmission line and the ground plane. We also adjusted the width of the transmission line to 2.5 mm to achieve the widest bandwidth, as shown in Fig. 2(b).

Figure 2(c) shows the prototypes of the realized electromagnetic radiator. The proposed antenna fabricated on an organic flexible substrate with thickness of 1.6 mm. The electromagnetic radiator has a rectangular patch with dimension of 14 × 18 mm^2^ and is fed by a microstrip line. A 50-ohm characteristics impedance was achieved with a 2.5 × 6 mm^2^ microstrip feed line. On the back side of the substrate, a fractional ground plane of side length (*Lg*) 3.5 mm was fabricated. We utilized the magnetron sputtering for copper coating of 200 nm thickness to fabricate the patch and ground plane of the electromagnetic radiator. The overall dimension of the antenna is 14 × 24 mm^2^, and for experimental verification, we fabricated a prototype of the proposed antenna.

The simulation of the proposed antenna was performed using high frequency simulation software (HFSS) based on the finite element method. In this simulation, we applied the obtained dielectric permittivity and loss tangent value to measure the reflection coefficient. The small height and low dielectric constant of the substrate reduced the undesired radiation and coupling, thereby improving the antenna performances. The thickness of the substrate of 1.6 mm helped to make the antenna light and also reduced the surface wave losses.

We employed the PNA network analyser of Agilent N5227A to measure the reflection coefficient (S11), transfer function (S21) and phase variation between antenna 1 and antenna 2, with and without a phantom. Later, we considered the electromagnetic performances of gain, efficiency and radiation pattern, both at flat and bending conditions, utilizing the near field anechoic chamber of SATIMO Star-Lab, as shown in Fig. 2(d) to (f).

In SATIMO, the electric field of the antenna are evaluated through near field measurement techniques, for which the data corresponds to the far field data, as the electromagnetic induction has the strongest forces in this field. This near field is known as the Fresnel region and can be described by ref. 26:


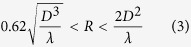


where *R* is the far field region, and *D* is the maximum linear dimension of the antenna. Later, this near field data are converted to far field data through Fourier transformation to portray the radiation pattern. The SATIMO near field system consists of the following: 16 identical and equally apart measurement probes that are positioned in a circular arc shape frame and a test board to hold the antenna in the centre and a network analyser. The radiation pattern has been measured, plotted and analysed using the combination of the 360° horizontally rotating values of the test antenna and an array of the 3D scan values of the probe. Afterwards, we calculated the gain and efficiency of the antenna from the far-field data of the radiation pattern.

**Figure 3:**
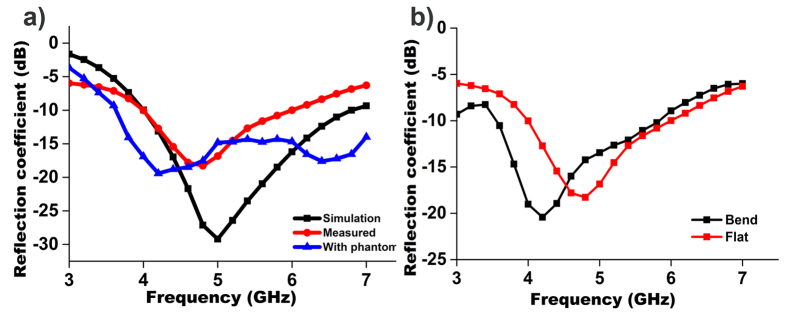
(**a**) Simulated and measured reflection coefficient of the proposed UWB flexible antenna with and without phantom; (**b**) measured reflection coefficient of the proposed UWB flexible antenna at both flat and bending condition.

Figure 3(a) and (b) show the measured (flat and bend) and simulated reflection coefficient versus frequency for the realized antenna. Measurements of the antenna were performed in the frequency range of 3 to 7 GHz. The measured resonant frequencies were close to the simulation ones. A small deviation was observed because of the fabrication tolerance.

To validate the on-body performance, the reflection coefficient was measured with the antenna in contact with the breast phantom. From Fig. 3(a), a negligible variation of the measured reflection coefficient was observed and the resonance shifted towards lower frequency. The main reason for this shift is the dielectric loading of the phantom. This variation did not effect the antenna performance during the detection of the tumour because the complete operational bandwidth was still below −10 dB.

Next, we tested the antenna bent downward at ±45° to conform with the breast phantom; the findings reveal that at bending condition the resonant frequency shifted towards lower frequency with higher impedance bandwidth, but remained within the operating band. The fabricated organic substrate exhibits significant flexibility and heftiness without any mechanical damage to the antenna.

**Figure 4:**
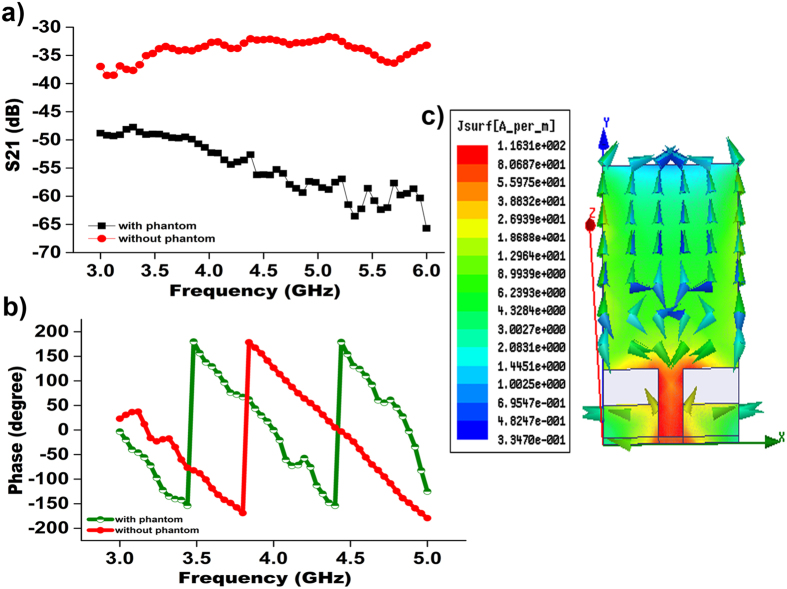
(**a**) Measured S21 amplitude between antenna 1 antenna 2 with and without phantom; (**b**) measured phase variation between antenna 1 and antenna 2 with and without phantom; and (**c**) surface current distribution of the proposed omnidirectional antenna.

**Figure 5:**
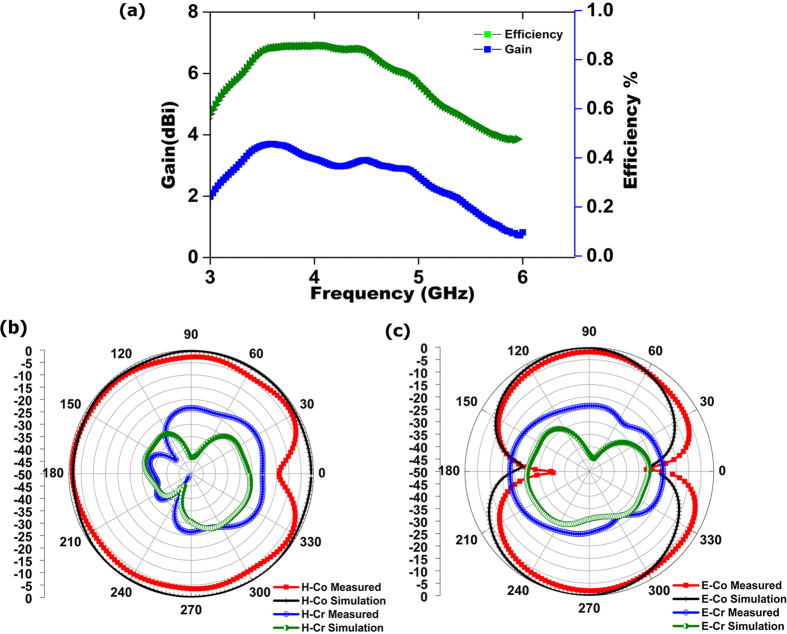
(**a**) Measured gain and efficiency of the proposed antenna at flat condition; (**b**) normalized H-plane radiation pattern of the proposed antenna; and (**c**) normalized E-plane radiation pattern of the proposed antenna.

The reflection coefficient (S11) magnitude is significant for impedance matching, while the transfer coefficient (S21) phase linearity is appropriate for a pulse based communication system. The measured S21 (transfer function) and phases results between antenna 1 and antenna 2 are plotted in Fig. 4(a) and (b). The transmission coefficient (S21) is recorded with the antennas separated by 20 cm and the apertures aligned. The phase variation of the proposed antenna was measured for the input impedance. Figure 4(b) demonstrates a linear

phase variation across the complete operating band. This linear variation in the phase with frequency ensures that all the frequency components of signal have same delay, leading to less pulse distortion. Lossy characteristics were observed in the presence of the phantom during the measurement of transfer function and phase variation. Figure 4(a) and (b) show that both a linear phase variation and an adequate transfer coefficient were obtained, even after introducing the dielectric loss. Some transmitted power is dissipated within the phantom because of the lossy material (high water content), resulting in the variation of S21 as illustrated in Fig. 4(a). Figure 4(c) shows the excited surface current distribution of the antenna. The vector current distribution shows that the current is flowing vertically without any nulls. This finding indicates that antenna is operating at its fundamental resonant mode, resulting in a dipole-like radiation pattern. From the figure, the surface currents are found to be aligned and equally distributed in the same direction. The current induced at the feed points of the matching circuit primarily contributes to the current distribution. Such current distribution in the vertical direction of the antenna generally creates an eight-shaped radiation pattern for the E plane and an omnidirectional radiation pattern for the H-plane, as shown in Fig. 5.

In a pulse based system, it is important that antenna radiates the pulse without any distortion. Any deviation in the transfer function will cause distortion of the signal. Therefore, the phase and the magnitude of the transfer function should be as linear as possible. The distortion of the signal of the proposed antenna into the breast tissue is evaluated by the fidelity factor. The fidelity factor is applied to measure the magnitude and phase transfer function of the antenna. If τ is the transmission delay, then the correlation factor is defined by the following equation^15^:


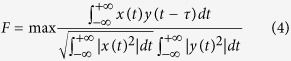


where the transmitted (TX) and received (RX) signal are represented as *x (t*) and *y (t*), respectively. For both face-to-face and side-by-side configurations, antennas are placed at a distance of 200 mm from each other. MATLAB is used to develop the fidelity equation and calculate its value. The fidelity factor is 0.8647 and 0.8057 for face-to-face and side-by-side configurations. The fidelity factor of the realized antenna in the face-to-face orientation is more novel than that of the recently published Vivaldi antenna. A higher degree of correlation confirms the lower distortion between transmitted and received antennas that is essential for ultra-wide band microwave imaging.

Figure 5(a) illustrates the realized gain for the proposed antenna at flat position. The figure shows the measured realized gain of the proposed antenna at the boresight (+z direction) in the operational frequency bandwidth. The electromagnetic radiators have a stable gain above 2 dBi throughout the operational frequency range. It is also observed that the variations are less than 1 dBi between flat and bending conditions.

In addition to the internal structural losses of the antenna, the total antenna efficiency (*ηT*) consists of matching losses at the input port, as given below^26^:


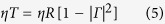


where *Γ* refers to reflection coefficient at the input port, and *ηR* denotes the radiation efficiency, which is the ratio of radiated power (PR) and power loss (PL) of the antenna^26^:


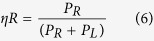


This power loss consists of the substrate dielectric loss and the radiation loss of the antenna. Therefore, the maximum radiation efficiencies refer to its total efficiency. Figure 5(a) depicts radiation efficiency is greater than 71% at flat conditions.

The simulated and measured radiation pattern at flat condition of the realized antenna is illustrated in Fig. 5 in 2D view for the two principal planes, named *H* plane and *E* plane. An omnidirectional radiation pattern was observed from the normalized *H* plane for co-polarization, with a dip near 0° and a donut-shape for cross polarization created by two nulls near 180° and 0°, as shown in Fig. 5(b). Moreover, a symmetric and stable radiation profile was realized. A Co-polar −3 dB half power beam width (HFBW) was measured for both flat and bending conditions. A total 304° HFBW was achieved in the *H* plane for the flat condition; in addition, no variation in the front lobe was observed for both cases, and a very negligible variation was observed near 0°.

A considerable amount of back lobe was observed, possibly because of the utilization of partial ground plane. A full ground plane can reduce this back lobe at the cost of low gain, low efficiency and narrow bandwidth. Figure 5(c) also shows the *E* plane radiation pattern. It is found that the proposed antenna produced a donut-shape radiation pattern resulting from nulls near 180° and 0° of the radiation pattern at the *E* plane; this pattern is consistent with the half wave dipole radiation pattern. A Co-polar −3 dB half power beam width (HFBW) for the *E* plane is 70° (58–128) and 102° (232–334). The difference between the simulation and measurement data are considered to be caused by the fabrication tolerance.

**Figure 6:**
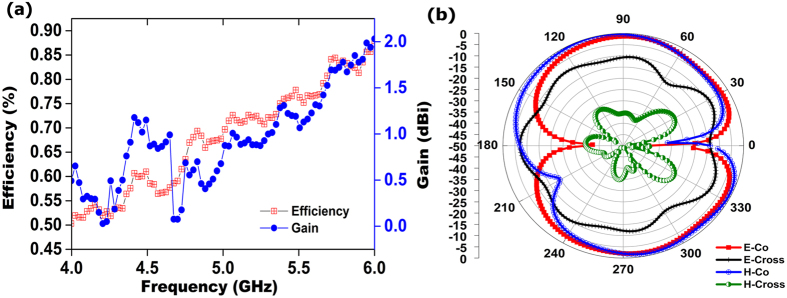
(**a**) Measured gain and efficiency of the proposed antenna at bending conditions and (**b**) normalized H-plane and E-plane radiation patterns at bending conditions.

To consider the antenna for a wearable bra in the future, the electromagnetic performances were examined at bending condition. The reflection coefficient of the antenna at bending conditions in Fig. 3(b) shows that although the resonant frequency shifted to the lower frequency, it still covers the required operational bandwidth. The proposed antenna at bending condition achieved an average efficiency above 70%, with an average realized gain of 1 dBi, as shown in Fig. 6(a). The measured radiation pattern of the realized antenna in bending conditions is presented in Fig. 6(b). The HPBW in the *H* plane from 20° to 126° and 232° to 344° construct an omnidirectional radiation pattern for the *H* plane. Two dips were observed near 0° and 210° for the *H* plane, possibly the result of the bending of the radiating element. For the *E* plane, a donut-shape radiation pattern resulting from two nulls in the broadside direction was observed; this pattern is similar to the typical monopole antennas for a copolarized field. Some dips for both the *E* plane and the *H* plane at cross polarization were observed as well because of the microstrip feed line printed directly below the ground plane. The stable radiation patterns, even at bending conditions, help the proposed flexible antenna to collect backscattering signal adequately and construct the image clearly.

## Imaging Set-up and Results

We applied a commercially available standard breast phantom from Japan in our experiment. Two nearly identical phantoms were used, with one embedded with a foreign object to mimic a cyst, whereas the other is completely homogeneous. The size of each phantom is approximately 16 × 8 cm^2^, and it is filled with standard dielectric constant as a human breast; the phantom with a foreign object also has a target tumour of 10 mm diameter inside it. The phantom considered in our examination consists of four layers, namely, the skin layer, the breast tissue layer or fat, the cancer benign breast tumour and the normal air layer. The dielectric permittivity and conductivity of the skin layer are 38 and 1.49 S/m, respectively, with a thickness of 2.5 mm. The breast tissue layer has a maximum width of 8.75 cm, with a conductivity of 0.141 S/m and dielectric permittivity of 5.14. The dielectric permittivity of the cancer benign breast tumour is 67^27^.

In our study, we utilized an automatic microwave imaging system consisting of two omnidirectional UWB antennas connected to the VNA (Agilent N-227A), a homogeneous breast phantom with a single tumour of higher dielectric constant, a mechanical rotation platform and a PC to control all the devices and electromechanical circuits. The transmitting antenna sends a microwave pulse to the examined breast, whereas the receiving antenna collects the backscattering signals that are reflected from the breast tissue; subsequently, the signals are analysed using a suitable computing system to determine whether a tumour is present. An ultra -wideband antenna in microwave imaging system can radiate a wide range of signals with adequate fidelity factor of the waveform within a large angular range to create precise images of high resolution. Before initiating the diagnosis procedure, the entire system was calibrated over the entire operating band (3–6 GHz) by utilizing the SOLT calibration kit (Agilent 85052 D 3.5 mm). This calibration in the imaging system will minimize the reflection at antenna-air interface. Once the calibration was completed, the antennas were gently placed both at flat and bending conditions on two opposite sides of the realistic breast phantom. We placed the tumorous phantom in a manner that the tumour is positioned 90° away from the starting point. Next, the phantom was rotated using an Arduino Uno circuit based stepper motor in the clockwise direction from 0° to 360° with a step size of 3°, resulting in 120 equivalent values. Afterwards, the controlling device triggered the VNA to start the measurement of the backscattering (S) parameters (S11, S21, S12 and S22) over the entire operating band and transmitted from the VNA to the PC by using the GPIB port. Subsequently, data were processed using the Delay-Multiply-and–Sum (DMAS) algorithm^14^ to reconstruct the image in the frequency domain, and an inverse discrete Fourier transform

(IDFT) was used to convert the S-parameters to the time domain as follows^28^:


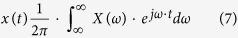


where *X (ω)* indicates the amount of signal at a given frequency contained in the original signal.

**Figure 7:**
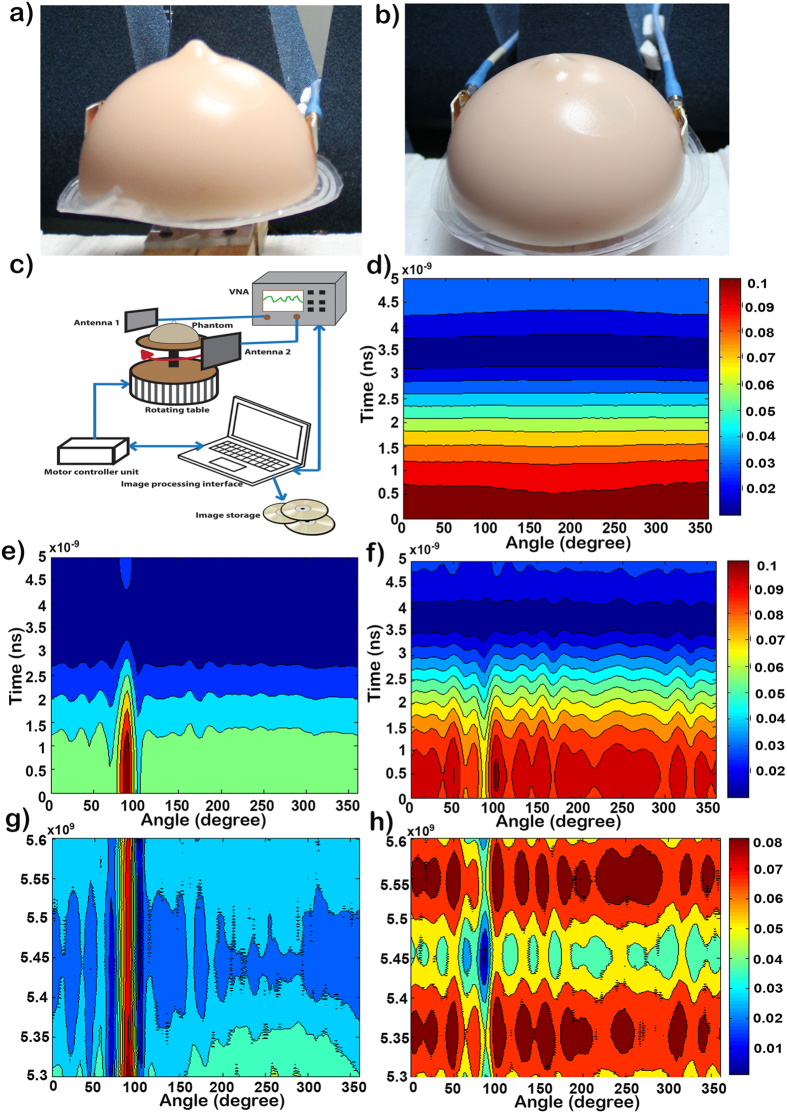
(**a**) Measurement setup with the antenna attached with the phantom skin; (**b**) antenna attached at bending conditions with the phantom skin; (**c**) Measurement setup for image processing; (**d**) time domain output without any cyst inside the phantom; (**e**) time domain output with cyst inside the phantom; (**f**) time domain output with cyst inside the phantom at bending conditions; (**g**) frequency domain output with cyst inside the phantom; and (**h**) the frequency domain output with cyst inside the phantom at bending conditions.

The DMAS algorithm reconstructed image highlights the electromagnetic scattering rather than recovering the dielectric property profile. This result shows a sharp image of the internal structure of breast phantoms. In this experiment, we focus on detecting the tumour only as an early stage of diagnosis, not the size and location of the tumour, as it will be the next step of diagnosis and treatment. Completion of the process takes only 5 minutes; the total process is shown in Fig. 7(a) to (c).

Figure 7(d) to (h) show the images of the breast tumour detected by the proposed antenna at both flat and bending conditions and presented in the frequency domain and the time domain. The reconstructed images with 120 data samples around the breast reveal that both antennas can be used to detect the tumour. It is evident that when we examined the healthy phantom, the signals are transferred through the breast tissue, and no changes were observed, as shown in Fig. 7(d). Afterwards, we examined the tumorous phantom, where our proposed antenna can clearly detect the tumour at 90° with the phantom positioned accordingly. The frequency domain results clearly show the presence of a cyst at approximately 90 degrees. The red vertical line in Fig. 7(g) indicates that there is a significantly higher return loss. The non-homogeneity caused by the cyst results in multiple interfaces at which reflections occur. These reflections cause the overall return loss to increase as more power is reflected to the antennas than dissipated within the breast phantom. In the time domain response, we observed that the reflected signal is from a source very close to the surface of the breast phantom. The centre of the reflected peak is below 0.5 ns, as shown in Fig. 7(h), which indicates that the cyst is just a few millimetres below the skin layer.

## Conclusion

Breast cancer is one of the deadliest cancers. Only regular diagnosis can reduce the mortality rate. However, women are rarely interested in such a diagnosis because of the uncomfortable process and high cost. Our research involves the development of a biodegradable and wearable antenna, namely, a “One time use clinical bra” to detect any growth of unwanted tissue inside the breast at an early stage. We chose an organic material as an antenna substrate so that it can be naturally degradable as well as flexible and cost effective. Moreover, electronic devices fabricated from renewable and biodegradable organic materials are desirable because frequently discarded electronic devices result in a serious environmental pollution problem. A bendable antenna that can easily be placed inside a bra has many advantages, e.g., low cost, ease of installation and fabrication, and light weight. Analytical and experimental results using a simulation model along with experimental measurements using a skin phantom were presented. A stable omnidirectional radiation pattern with minimum average radiation efficiency above 70% and minimum average realized gain beyond 1 dBi were achieved over the operating frequency range at bending conditions. In conclusion, the optimal performances of the proposed flexible antenna validate its potential as a suitable candidate for use in microwave breast imaging based on a wearable medical bra in the near future.

## Methodology

The organic compound was prepared using conventional synthesis procedures from 4-(perfluorohexyl)bromobenzene with corresponding 5-formyl-2-thiophene broronic acid as light yellow solid. After purifying the compound by silica gel column chromatography (60 N spherical, neutral, 40–100 mm) using dichloromethane and hexane (1:1) solvent as eluent, ^1^H NMR (BRUKER 400 MHz), ^13^C NMR (BRUKER 400 MHz), and mass spectrometry were conducted to confirm the molecular structure and purity. ^1^H NMR spectra are reported as follows: chemical shift in ppm (d) relative to the chemical shift of Chloroform-d (CDCl_3_) at 7.26 ppm, integration, multiplicities (s = Singlet, d = Doublet, m = Multiple): d 9.94 (s, 1 H), 7.82–7.79 (m, 2 H), 7.68–7.67 (d, 2 H) and 7.51–7.50 (d 2 H); ^13^C NMR (400 MHz, CDCl_3_) spectra are reported in ppm (d) relative to the chemical shift of Chloroform-d (CDCl3) at 77.0 ppm and the chemical shifts of are as follows: d 182.7, 151.7, 143.8, 137.1, 136.6, 129.7, 129.5, 127.9, 127.8, 127.8, 126.6 and 125.4; The m/z: Calcd for C17H7F13OS 506.0; and found: 506.0. This organic compound is stable under moisture and oxygen and is a heterocyclic compound (single molecule) that can be decomposed under desired conditions. Such organic materials are encouraged for applications in electronic devices to minimize the occupational and environmental hazards and promote economic benefits.

## Additional Information

**How to cite this article**: Rahman, A. *et al*. Electromagnetic Performances Analysis of an Ultra-wideband and Flexible Material Antenna in Microwave Breast Imaging: To Implement A Wearable Medical Bra. *Sci. Rep.* **6**, 38906; doi: 10.1038/srep38906 (2016).

**Publisher's note:** Springer Nature remains neutral with regard to jurisdictional claims in published maps and institutional affiliations.

## Figures and Tables

**Table 1:** Comparison between various breast cancer detection methods^8^.

Modality	Sensitivity	Specificity	Positive Predictive Value	Accuracy
Mammography	67.8% (120/177)	75% (61/81)	85.7% (120/140)	70.2% (181/258)
Mammography and clinical examination	77.4% (137/177)	72% (58/81)	58.6% (137/160)	75.6% (195/258)
Clinical examination	50.3% (89/177)	92% (75/81)	94% (89/95)	63.6% (164/258)
Ultrasound	83% (147/177)	34% (28/81)	73.5% (147/200)	67.8% (175/258)
Mammography and ultrasound	91.5% (162/177)	23% (19/81)	72.3% (162/224)	70.2% (181/258)
Mammography, clinical examination and ultrasound	93.2% (165/177)	22% (18/81)	72.4% (165/228)	70.9% (183/258)
MRI	94.4% (167/177)	26% (21/81)	73.6% (167/227)	72.9% (188/258)
Mammography, clinical examination, and MRI	99.4% (176/177)	7% (6/81)	70.1% (176/251)	70.5% (182/258)
